# Therapeutic Effects of Fermented Flax Seed Oil on NC/Nga Mice with Atopic Dermatitis-Like Skin Lesions

**DOI:** 10.1155/2017/5469125

**Published:** 2017-01-19

**Authors:** Joonhyoung Yang, Sangyeon Min, Seungug Hong

**Affiliations:** ^1^Department of Ophthalmology & Otolaryngology & Dermatology, College of Korean Medicine, Dongguk University, Dongguk-ro 27, Ilsandong-gu, Goyang-si, Gyeonggi-do 10326, Republic of Korea; ^2^Department of Pediatrics, College of Korean Medicine, Dongguk University, Dongguk-ro 27, Ilsandong-gu, Goyang-si, Gyeonggi-do 10326, Republic of Korea

## Abstract

*Background*. Atopic Dermatitis (AD) is one of the most common chronic inflammatory skin diseases.* Objective*. This experiment aimed to study the effects of Fermented Flax Seed Oil (FFSO) on symptoms such as redness, eczema, and pruritus induced by AD.* Materials and Methods*. AD-induced NC/Nga mice were used to observe the immunological and therapeutic effects of FFSO on skin in vivo. Raw 264.7 cells were used to investigate the effects of FFSO in cells. Fc receptor expression and concentration of beta-hexosaminidase were measured. Nitric oxide assay, Western blotting, real-time PCR, image analysis, and statistical analysis were performed in vitro.* Results*. In the immunohistochemical results, p-ERK 1/2 expression decreased, fibrogenesis strongly increased, and distribution reduction is observed. Distribution of IL-4-positive cells in the corium near the basal portion of the epithelium in the AT group was reduced. FFSO treatment reduced the number of cells showing NF-*κ*B p65 and iNOS expression. The level of LXR in the AT group was higher than that in the AE group, and elevation of PKC expression was significantly reduced by FFSO treatment.* Conclusion*. FFSO could alleviate symptoms of AD such as epithelial damage, redness, swelling, and pruritus.

## 1. Introduction

Atopic Dermatitis (AD) is one of the most common chronic inflammatory skin diseases and is caused by environmental pollution due to industrial development in modern society. According to several reports, AD usually occurs in about 20% of children and 1~3% of adults [1–3].

The most common symptoms of AD are skin redness, pruritus, infection, and lichenification. According to recent studies, the latest pathological view of AD is that abnormal differentiation of skin epithelial cells causes skin barrier impairment, after which extrinsic stimulatory factors induce immune responses in the impaired part and the systemic immune response is activated [4–6].

Most previous studies related to skin barrier function have focused on the causes of skin barrier abnormalities, as an initial factor of AD, due to lipid reduction between keratin cells [7, 8].

In previous studies, perilla and flax seeds were reported to contain high contents of lipids similar to keratin cells in the skin [9, 10]. Flax seeds are relatively cheaper and easier to preserve than perilla seeds. Therefore, flax seeds were used in this experiment.

However, like other seeds, flax seeds contain infinitesimal amounts of cyanide glycoside, which can give rise to toxicity when used long-term or overdosed [11]. To avoid toxicity, we extracted oil by grinding flax seeds after lactic acid fermentation processing.

We observed the in vivo immunological and therapeutic effects of Fermented Flax Seed Oil (FFSO) on the skin of AD-induced NC/Nga mice. We also investigated the in vitro effects of FFSO in Raw 264.7 cells.

This experiment aimed to study the effects of FFSO on symptoms such as redness, eczema, and pruritus induced by AD.

## 2. Materials and Methods

### 2.1. *Lactobacillus* Cultivation

We were provided a mixture of 12 kinds of Alpha* Lactobacillus* (*Lactobacillus acidophilus*,* L. bulgaricus*,* L. casei*,* L. fermentum*,* L. paracasei*,* L. plantarum*,* L. rhamnosus*,* L. reuteri*,* Bifidobacterium bifidum*,* B. breve*,* B. longum*, and* Streptococcus thermophilus*, each 1.0 × 10^10^ CFU/g or more) by EM life science research institute. We used a mixture of 12 kinds of Alpha* Lactobacillus* by diluting 5 grams of* Lactobacillus* with 50 mL of mineral water.

### 2.2. Manufacturing and Processing of Fermented Flax Seeds

We purchased roasted flax seeds, which were imported from Canada, from Jeongudang (http://www.herbseoul.com/). After grinding 500 grams of flax seeds, we spread* Lactobacillus* diluents by spraying. The ground flax seeds were fermented at 40°C for 48 hours and then dried. We obtained 204 mL of extracted flax seed oil by pressing. The FFSO was administered to mice at a concentration of 0.2 g/kg per day.

### 2.3. Animal and AD Induction

Male 6-week-old NC/Nga mice (18–20 g each) were obtained from Central Lab Animal Inc. (Seoul, Korea) and maintained in an air-conditioned animal room for 2 weeks before the experiments. The mice were divided into three groups (*n* = 10 per group) as follows: untreated mice (Ctrl group), AD-induced mice with no treatment (AE group), and FFSO treated AD-induced mice (AT group). To induce AD-like skin lesions, back regions of mice were stripped, and 1 mL of 5% sodium dodecyl sulfate (SDS) (Sigma-Aldrich, St. Louis, MO, USA) was rubbed onto the back of each mouse 20 times using a cotton swab to remove the lipid lamella of the stratum corneum.* Dermatophagoides *(D.)* farinae* crude extract (100 mg, Biostir Inc., Kobe, Hyogo, Japan) was applied six times per week for 3 weeks. Mice in the AT group were given orally 0.2 g/kg of FFSO for 3 weeks daily. Animals were sacrificed 72 h after the last* D. farinae* application, and histological specimens were collected. All procedures involving animals were approved by the Institutional Animal Care and Use Committee of Dongguk University (IACUC number: DGU-2014-*∗∗∗∗*). We followed the NIH Guide for the Care and Use of Laboratory Animals throughout this study. All data were evaluated by observers who were blinded to the experimental conditions.

### 2.4. Histochemistry

Sodium pentobarbital was used to sedate mice. Anesthetized mice were fixed in 10% neutral-buffered formalin (NBF) solution, and a vascular rinse was performed for 24 hours at room temperature. Fixed dorsal tissue slices were embedded in paraffin. After embedding, 5 *μ*m thick sections were histochemically stained. To investigate changes in capillaries such as size and branching, we performed Phloxine-tartrazine staining. To investigate epidermal changes such as collagen fiber distribution, we performed Masson's trichrome staining. To investigate the distribution and morphological changes of mast cells, we performed histochemical staining with Luna's stain. To investigate the lipid lamella in the epithelium, we performed histochemical staining with Oil-red O after samples were prepared according to routine historical procedures for cryosectioning [12].

### 2.5. Immunohistochemistry

The skin slices were steeped in proteinase K solution (20 *μ*g/mL) to undergo proteolysis for 5 minutes. The proteolyzed slices were incubated in blocking serum (10% normal goat serum) for 4 hours, after which slices were incubated with the primary antibodies goat anti-p-ERK 1/2 (1 : 100, Santa Cruz Biotec, USA), goat anti-Fc *ε* receptor (1 : 100, Santa Cruz Biotec), goat anti-substance-P (1 : 100, Santa Cruz Biotec), goat anti-MMP-9 (1 : 100, Santa Cruz Biotec), goat anti-5HT (1 : 100, Santa Cruz Biotec), goat anti-TNF-*α* (1 : 250, Santa Cruz Biotec), goat anti-NF-*κ*B p65 (1 : 500, Santa Cruz Biotec), goat anti-iNOS (1 : 250, Santa Cruz Biotec), goat anti-COX-2 (1 : 100, Santa Cruz Biotec), goat anti-LXR (1 : 200, Santa Cruz Biotec), goat anti-PKC (1 : 100, Santa), goat anti-IL4 (1 : 100, Santa Cruz Biotec), goat anti-STAT 6 (1 : 100, Santa Cruz Biotec), goat anti-IL6 (1 : 100, Santa Cruz Biotec), and goat anti-STAT 3 (1 : 100, Santa Cruz Biotec) for 72 hours in a 4°C humidified chamber. Next, the slices were linked with biotinylated rabbit anti-goat IgG (1 : 100, Santa Cruz Biotec), which is a secondary antibody, for 24 hours at 4°C. The biotin secondary antibody-linked specimens were conjugated with Avidin (Avidin biotin complex kit, Vector Lab, USA) for 1 hour at room temperature. Finally, the slices were developed with 0.05% diaminobenzidine and then nuclear counter-stained with hematoxylin.

### 2.6. Fc Receptor Expression

We measured the effects of FFSO samples on gene expression of Fc receptor on the surface of Raw 264.7 cells. A total of 4 × 10^5^ Raw 264.7 cells were seeded in a 6-well plate, and the six FFSO samples were treated for 4 hours. FFSO samples were then subjected to cDNA synthesis and real-time PCR. The results show the ratio of Fc receptor to GAPDH expression in cells.

### 2.7. Beta-Hexosaminidase

We measured the concentration of beta-hexosaminidase in both the supernatant and the cells. Briefly, RBL-2H3 cells were seeded in 6-well plates at a concentration of 3 × 10^5^ cells/mL, and FFSO samples were treated for 1 hour. We treated each FFSO sample with 50 nM PMA and 1 *µ*M A23187, followed by culture at 37°C for 50 minutes in an incubator. After collecting the supernatant and cell pellet separately, we added 50 *µ*L of 1 mM *ρ*-nitro-phenyl-N-*β*-D-glucosaminide by melting 0.1 M citrate buffer (pH 5) in 50 *µ*L supernatant in each well. After incubation at 37°C for 1 hour, the reaction was terminated by treatment with 200 *µ*L/well of 0.1 M carbonate buffer (pH 10.5). The absorbance of the reactant was read at a wavelength of 405 nm in a 96-well plate. The inhibition percentage of secretion was calculated using the following equation. A405 is absorbance of the reactant measured at a wavelength of 405 nm, sup is supernatant, and pellet is cell layer. This experiment was performed in triplicate.

(1) Beta-hexosaminidase  release  %=A405  of  supA405  of  Sup+A405  of  pellet∗100.

### 2.8. Nitric Oxide Assay

The effect of FFSO treatment on NO secretion by Raw 264.7 cells was measured in a color reaction using Griess reagent and cell supernatant. Raw 264.7 cells were seeded in 6-well plates (4 × 10^5^ cells/well) and incubated for 24 hours. After FFSO pretreatment at various concentrations for 6 hours, LPS was treated at a concentration of 1 microg/mL and incubated for 24 hours. Supernatant samples without the cell layer were mixed with the same volume of Griess reagent mixture and reacted at room temperature for 10 minutes. The reactant was transferred to a 96-well plate, and the absorbance was measured at a wavelength of 540 nm. The reactant was quantified using sodium nitrite at various concentrations. This experiment was performed in triplicate.

### 2.9. Western Blotting

The expression ratios of COX-2 and iNOS, which are inflammatory factors, compared to beta-actin were determined. NF-kB translocation from the cytoplasm to karyoplasm (nucleoplasm) was determined by comparing the amounts of protein in dermal papilla cells and lamin B. Cox-2 and iNOS expressions were pretested with various concentrations of FFSO in Raw 264.7 cells (4 × 10^5^ cells) for 30 minutes, after which LPS was treated at a concentration of 1 microg/mL for 24 hours. NF-kB was pretreated with flax seed oil for 2 hours, after which the same concentration of LPS was treated for 1 hour. Cell lysate was produced from the harvested cells using RIPA buffer and NE-PER nuclear and cytoplasmic extraction reagent was used for extracting nuclear proteins according to manufacturer's instruction. In brief, Raw 264.7 cells were appropriately washed with DPBS and harvested. Cells were added with CER I reagent for 10 min, following CER II reagent addition and incubation for one minute. Cells were vortexed and centrifuged at maximum speed (16,000*g*) for 5 min to transfer the supernatant containing cytoplasmic proteins. Pellets containing nuclei were added with NER and vortexed at every 10 min for total 40 min. Lysates were centrifuged at maximum speed (16,000*g*) for 10 min and supernatant fraction containing nucleus protein was transferred to new tube. Each protein concentration of fractionation was measured separately to be used in Western blotting. Protein concentration was measured by using the BCA method. The absorbance was measured at 562 nm using various concentrations of BSA as a standard. After loading the same amounts of protein onto a 10% SDS-PAGE gel, we performed gel electrophoresis at 120 v for 100 minutes, followed by transfer at 130 v for 90 minutes to a PVDF membrane. The membrane was then blocked with melted 5% skim milk and BSA in TBST for 30 minutes. Upon antibody binding to the membrane, the primary antibody was shaken with 3% skim milk and BSA overnight. The secondary antibody was incubated with 1% skim milk and BSA for 1 hour. We measured quantity of expression by ECL through the image using RAS-3000 (Fujifilm, JAPAN) after 2 minutes of exposure. This experiment was performed in triplicate.

### 2.10. Real-Time Reverse Transcription-Polymerase Chain Reaction (Real-Time PCR)

We compared the expression levels of COX-2, IL-6, IL-1b, and TNF-a with the expression level of GAPDH using the real-time quantitative PCR method.

Raw 264.7 cells were seeded in a 6-well plate (4 × 10^5^ cells/well), after which each FFSO sample was treated for 30 minutes. After treatment, samples were induced to undergo inflammation by treatment with 1 microg/mL of LPS. We eliminated cell supernatant after 12 hours and isolated RNA from cells with Trizol Reagent. After RNA isolation, we synthesized complementary DNA (cDNA) using AccuPower® RT PreMix reverse transcriptase PreMix. We arranged SYBR green Master Mix, primer with possible RNA hybridization in each gene, ultrapure water, and cDNA in each well of a 96-well PCR plate. We performed PCR the amplification using the Roche LightCycler LC480 instrument. This experiment was performed in duplicate.

### 2.11. Image Analysis and Statistical Analysis

To produce numerical data from our immunohistochemistry, image analysis was performed using image Pro Plus (Media cybernetics, USA). In the image analysis of our 400x magnification exposure photograph, positive reacted pixel cells (80–100 intensity range) were counted in 10 randomly selected fields of each group (total pixel cells 10,000,000). The data were presented as the mean ± SE. Statistical significance of the differences was analyzed with SPSS software (SPSS 23, SPSS Inc., USA), using a one-way ANOVA and Levene's (LSD) test with a significance level of *p* < 0.005.

## 3. Results

### 3.1. Alleviation Effects of AD-Induced Skin Lesions

**Figure 1:**
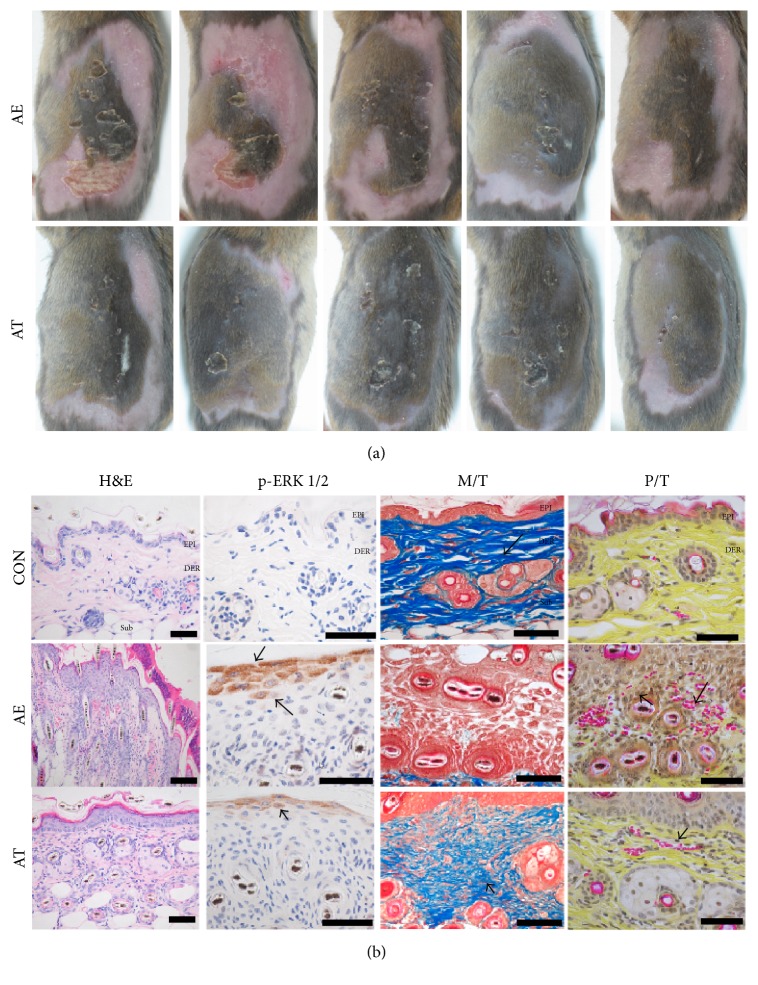
The mitigative effect of FFSO treatment for AD. (a) The skin damage as eczema was mitigated in AT. (b) The damage of intercellular space of stratum corneum, hyperplasia, and infiltration of lymphocytes were increased in AE but decreased in AT. (H&E satin, Bar size, 100 *μ*m). The p-ERK 1/2 positive reaction in the AT was decreased in stratum granulosum. (p-ERK 1/2 immunohistochemistry, Bar size, 100 *μ*m). The decrease of edema was shown in AT as compared with AE. (Masson's trichrome stain, Bar size, 100 *μ*m). The distribution of capillary was decreased in AT as compared with AE (Phloxine-tartrazine stain, Bar size, 100 *μ*m).* Abbreviations*. EP: epidermis; DE: dermis; M/T: Masson's trichrome stain; P/T: Phloxine-tartrazine stain; Ctrl, induced AD and given water; AE, induced AD and given water; AT, induced AD with FFSO treatment.

The therapeutic effects of FFSO were estimated based on external and histological morphologic changes in dermatitis severity. The AE group showed the highest level of dermatitis severity. The external shape of the AE group showed various pathological features, such as severe erythema, blood clot, edema, superficial erosion, deep excoriation, and dry skin. In contrast, AT ameliorated AD symptoms (Figure 1(a)).

For H&E staining, atopic mice of the AE group exhibited significant damage to the intercellular space of the stratum corneum, hyperplasia, edema, infiltration of fibroblasts, and an increased capillary distribution. On the other hand, in mice treated with FFSO, remarkable reduction of histological skin lesions was observed (Figure 1(b)).

For the immunohistochemical results, expression of p-ERK 1/2, a cell activator, was observed in the AE group in the stratum granulosum. Expression of p-ERK 1/2 in the AT group was reduced by 60% (*p* < 0.005) as compared with the AE group (Figure 1(b), Table 1).

Masson's trichrome staining was used to examine the variable deposition of collagen fibers at lesion skin sites. Results show the lower deposition of collagen fibers in the papillary and reticular layers of the dermis in the AE group. On the other hand, mice treated with FFSO showed a remarkable increase in fibrogenesis (Figure 1(b)).

Distributional comparison of capillaries was conducted between the AE and AT groups through Phloxine-tartrazine staining. Our results reveal that the AT group experienced much better distribution reduction than the AE group (Figure 1(b)).

### 3.2. Regulation of Mast Cell Activation

**Figure 2:**
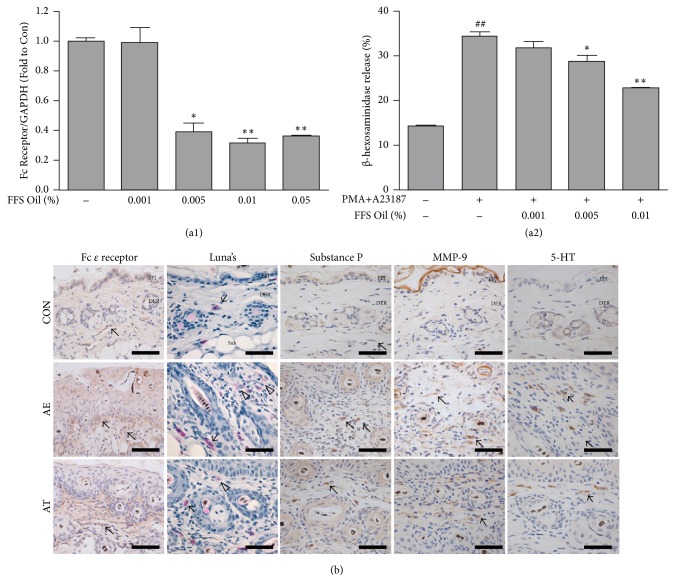
Regulation of mast cell activation. (a1) FFS Oil, FFSO; Ratio of Fc receptor to GAPDH expression in cells was determined by real-time PCR. Real-time PCR product-stimulated macrophages were analyzed as described in Section 2. Data were expressed as the mean ± SD of duplicate experiments in three different wells. (a2) Cells were treated with the indicated concentration of FFSO. Degranulation was assessed by *β*-hexosaminidase release into the supernatant. *β*-hexosaminidase released into the medium is presented as the mean ± SE. (b) Numbers of degranulated mast cell (white arrow) in the dermal papillae increased in the AE group but decreased in the AT group (Luna's method; Bar size, 50 *µ*m). Fc *ε* receptor-positive cells (arrow indicates dark brown) in the HTT remarkably decreased (substance-P immunohistochemistry; Bar size, 50 *µ*m). Mast cells secreting substance-P, MMP-9, and 5-HT (arrow indicates dark brown) in the AT group remarkably decreased (immunohistochemistry; Bar size, 50 *µ*m). MMP-9: matrix metalloproteinases-9, 5-HT: serotonin. Other abbreviations are the same as in Figure 1. ^*∗*^*P* < 0.05 compared with AE; ^*∗∗*^*P* < 0.01 compared with AE; ^##^*P* < 0.01 compared with Ctrl.

*(A) Cell Line Experiment*. Raw 264.7 cells were treated with FFSO at various concentrations. RNA was isolated from cells and then subjected to cDNA synthesis and real-time PCR. The results show that the ratio of Fc receptor to GAPDH expression in cells significantly decreased depending on the concentration. The ratio of Fc receptor expression decreased by 1% at 0.001% concentration, 60% at 0.005% concentration, 78% at 0.01% concentration, and 64% at 0.05% concentration (Figure 2(a1)).

We measured degranulation of RBL-2H3 mast cells by using *β*-hexosaminidase assay. Degranulation of PMA plus A23187-induced RBL-2H3 cells decreased in a FFSO concentration-dependent manner. Compared to the control group, degranulation decreased by 3% at 0.001% concentration, 15% at 0.005% concentration, and 34% at 0.01% concentration (Figure 2(a2)).

*(B) Animal Experiment*. Regarding Luna's staining results, many degranulated mast cells from the dermal papilla to the area around the subcutaneous layer were observed in the AE group. On the other hand, reduced numbers of degranulated mast cells were observed in the AT group as compared with the AE group (Figure 2(b)).

Regarding the immunohistochemistry results, Fc *ε* receptor expression increased in the AE group in the dermis. Fc *ε* receptor expression in the AT group decreased by 58% (*p* < 0.005) as compared with the AE group (Figure 2(b), Table 1).

Regulation of mast cell activation was estimated by measuring positive reactions for substance-P, matrix metallopeptidase 9 (MMP-9), and 5-HT. Marked increases in positive reactions for substance-P, MMP-9, and 5-HT were observed in the cytoplasm of dermal papilla cells in the AE group. Treatment with FFSO significantly suppressed elevation of these levels. Levels of substance-P in the AT group were reduced by 86% (*p* < 0.005) as compared with the AE group. Levels of MMP-9 in the AT group were reduced by 69% (*p* < 0.005) as compared with the AE group. Levels of 5-HT in the AT group were reduced by 85% (*p* < 0.005) as compared with the AE group (Figure 2(b), Table 1).

### 3.3. Downregulation of Inflammation

**Figure 3:**
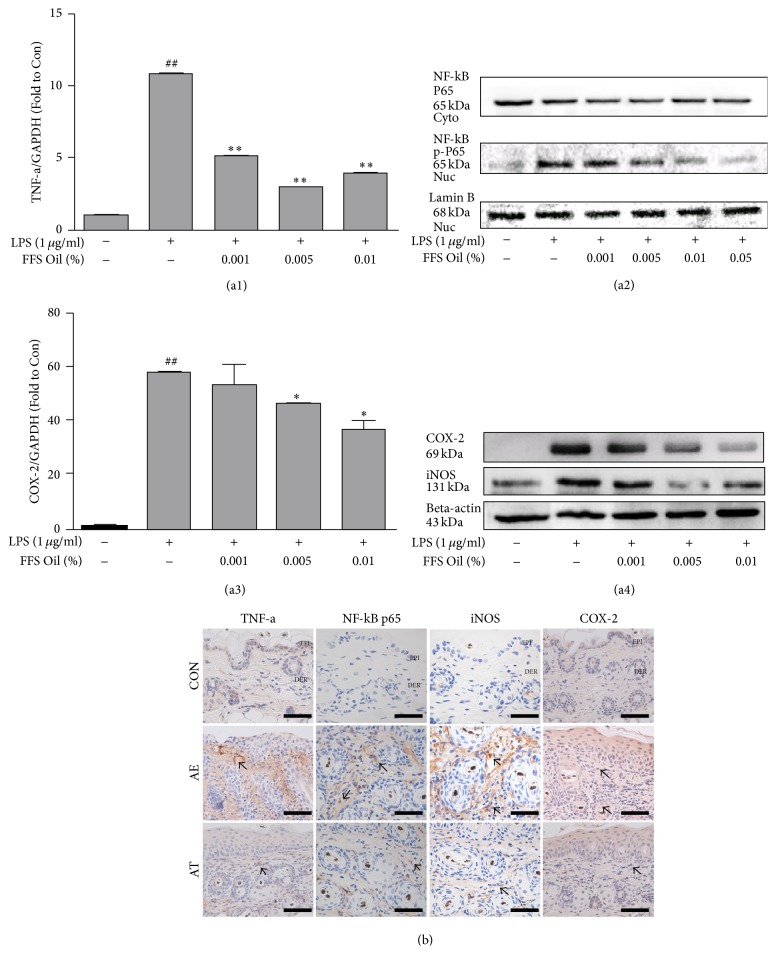
Downregulation of inflammation. (a1) Cells were pretreated with different concentrations of FFSO for 30 minutes and then stimulated with LPS (1 microg/mL) for 12 hours. Ratio of TNF-*α* to GAPDH expression in cells was determined by real-time PCR, as described in Section 2. (a2) Cells were pretreated with the indicated concentrations of FFSO for 2 hours and then stimulated with LPS (1 microg/mL) for 1 hour, after which nuclear extracts were prepared. Detection of NF-*κ*B-binding activities was performed as described in Section 2. This experiment was repeated two times with similar results. (a3) Ratio of COX-2 to GAPDH expression on cells same as (a1). (a4) Cells were pretreated with different concentrations of FFSO for 30 minutes and then stimulated with LPS (1 microg/mL) for another 24 hours. Expression of iNOS and COX-2 proteins was detected by Western blotting using specific anti-iNOS and anti-COX-2 antibodies. *β*-Actin protein was used as an internal control. Expression of TNF-*α*, NF-kB p65, iNOS, and COX-2 (arrow indicates dark brown) was induced by* D. farinae* in the AE group. These positive reactions in the AT group were remarkably decreased compared with those in the AE group (immunohistochemistry; Bar size, 50 *μ*m). Abbreviations are the same as in Figure 1. ^*∗*^*P* < 0.05 compared with AE; ^*∗∗*^*P* < 0.01 compared with AE; ^##^*P* < 0.01 compared with Ctrl.

*(A) Cell Line Experiment*. Raw 264.7 cells were treated with FFSO at various concentrations. RNA was isolated from cells and then subjected to cDNA synthesis and real-time PCR. We measured the ratio of TNF-*α* to GAPDH expression in cells. The results show that the ratio of TNF-*α* to GAPDH expression in cells remarkably decreased depending on the concentration. Compared to the control group, the ratio of TNF-*α* expression decreased by 54% at 0.001% concentration, 72% at 0.05% concentration, and 64% at 0.01% concentration (Figure 3(a1)). Likewise, we measured the ratio of COX-2 expression. Compared to the control group, COX-2 expression decreased by 8% at 0.001% concentration, 20% at 0.05% concentration, and 36% at 0.01% concentration (Figure 3(a3)). We also tested the effect of FFSO on LPS-induced nuclear translocation of p65 and phosphorylation of p65 by Western blotting. As shown in Figure 3(a2), FFSO suppressed nuclear translocation of the p65 subunit of NF-kB and nuclear phosphorylation of p65. Pretreatment with FFSO for 30 minutes reduced LPS-induced iNOS and COX-2 expression in a concentration-dependent manner (Figure 3(a4)).

*(B) Animal Experiment*. To estimate the anti-inflammatory effects of FFSO, we measured expression levels of TNF-*α*, NF-kB p65, iNOS, and COX-2. Immunohistochemical staining confirmed the expression of TNF-*α*, NF-kBp65, iNOS, and COX-2 in dermal papilla cells. Compared with AE, AT treatment significantly reduced expression levels of TNF-*α*, NF-kB p65, iNOS, and COX-2. The AE group showed a 66% (*p* < 0.005) decrease in TNF-*α* expression as compared with the AT group. The AE group showed an 86% (*p* < 0.005) decrease in NF-kB p65 expression as compared with the AT group. The AE group showed a 25% (*p* < 0.005) decrease in iNOS expression as compared with the AT group. The AE group showed a 65% (*p* < 0.005) decrease in COX-2 expression as compared with the AT group. (Figure 3(b), Table 1).

### 3.4. Regulation of Th2 Differentiation

**Figure 4:**
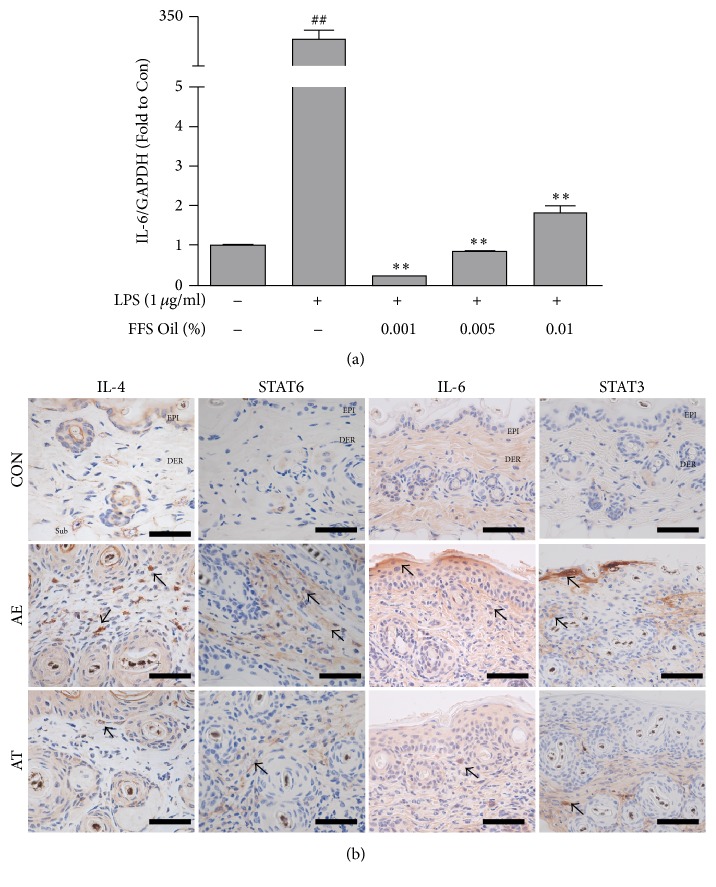
Regulation of Th2 differentiation. (a) Ratio of IL-6 to GAPDH expression in cells was determined by real-time PCR. Real-time PCR product-stimulated macrophages were analyzed as described in Section 2. Data were expressed as the mean ± SD of two separate experiments. (b) Expression of IL-4 and IL-6 (arrow indicates dark brown) in cells decreased in the AT group compared with the AE group (IL-4 and IL-6 immunohistochemistry; Bar size, 50 *µ*m). EP: epidermis, DE: dermis. STAT6 and STAT3 expression (arrow indicates dark brown) in cells decreased in the AT group compared with the AE group (STAT6 and STAT3 immunohistochemistry; Bar size, 50 *µ*m). Abbreviations are the same as in Figure 1. ^*∗∗*^*P* < 0.01 compared with AE; ^##^*P* < 0.01 compared with Ctrl.

*(A) Cell Line Experiment*. Raw 264.7 cells were treated with various concentrations of FFSO. RNA was isolated from cells, which were induced to undergo inflammation by treatment with 1 microg/mL LPS. We then measured the ratio of IL-6 expression by cDNA synthesis and real-time PCR. The results show that the ratio of IL-6 expression in cells remarkably decreased regardless of concentration. The ratio of IL-6 expression was similar to that in normal cells regardless of concentration. Compared to the control group, the ratio of IL-6 expression decreased by 99.9% at 0.001% concentration, 99.7% at 0.05% concentration, and 99.4% at 0.01% concentration (Figure 4(a)).

*(B) Animal Experiment*. Regulation of Th2 differentiation was estimated by measuring IL-4- and IL-6-positive reactions. IL-4-positive reaction was observed in the cytoplasm of dermal papilla cells. Levels of IL-4 in the AE group were decreased by 75% (*p* < 0.005) as compared with the AT group. IL-6-positive reaction was observed in the cytoplasm of dermal papilla cells. Levels of IL-6 in the AE group were reduced by 54% (*p* < 0.005) as compared with the AT group (Figure 4(b), Table 1).

Regulation of the JAK-STAT pathway for production of IL-4 and IL-6 was estimated by measuring STAT6- and STAT3-positive reactions. STAT6-positive reaction was observed in the cytoplasm of dermal papilla cells. Levels of STAT6 in the AE group were reduced by 94% (*p* < 0.005) as compared with the AT group. STAT3-positive reaction was observed in the cytoplasm of dermal papilla cells. Levels of STAT3 in the AE group were reduced by 67% (*p* < 0.005) as compared with the AT group (Figure 4(b), Table 1).

### 3.5. Maintenance of Lipid Barrier in Epidermis

Protective effects of lipid barriers were estimated by measuring liver X receptor- (LXR-) and PKC-positive reactions, which are known to be involved in lipid metabolism.

**Figure 5:**
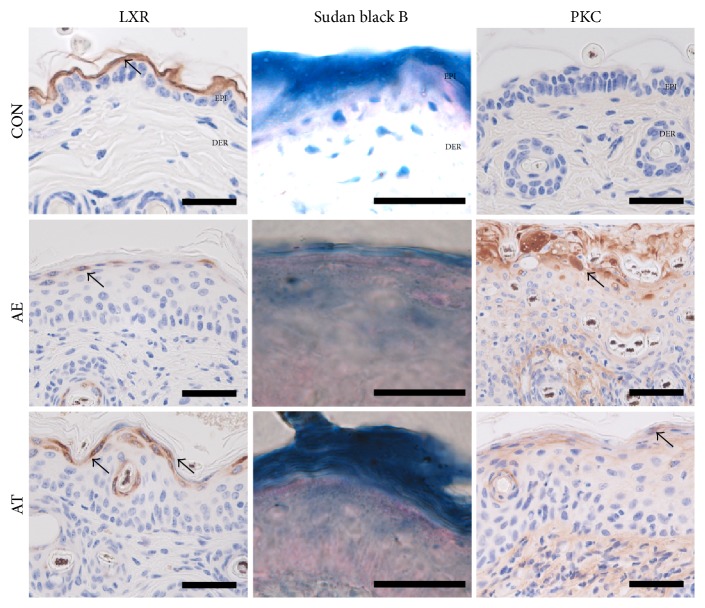
Maintenance of lipid barrier in epidermis. LXR expression (arrow indicates dark brown) remarkably decreased in the AE group but was maintained in the AT group (LXR immunohistochemistry; Bar size, 50 *µ*m). The lipid barrier (arrow indicates bright blue) in the intercellular space from the stratum granulosum to stratum corneum was absent in the AE group but present in the AT group (Sudan black B; Bar size, 50 *µ*m). PKC expression (arrow indicates dark brown) in the AE group remarkably decreased (PKC immunohistochemistry; Bar size, 50 *µ*m). LXR: liver X receptor, PKC: Protein kinase C; other abbreviations are the same as in Figure 1.

Levels of LXR in the cytoplasm of cells in the stratum corneum were remarkably reduced in the AE group, whereas levels of AT were elevated by 149% (*p* < 0.005) as compared with the AE group (Figure 5, Table 1).

Moreover, we observed that skin damage, such as elimination of the intercellular lipid lamellae in the stratum corneum, was remarkably reduced in the AT group as compared with AE group (Figure 5).

Increased positive reactions for PKC were observed in damaged keratinocytes and the intercellular space in the AE group. This elevation was significantly reduced by FFSO treatment. Levels of PKC in the AE group were reduced by 69% (*p* < 0.005) as compared with the AT group (Figure 5, Table 1).

## 4. Discussion

In this experiment, AD-induced NC/Nga mice were used. To induce AD-like skin lesions, back regions of mice were stripped, and SDS was rubbed onto the back of each mouse 20 times using a cotton swab to remove the lipid lamella of the stratum corneum by Christophers and Mrowietz's method [13].* Dermatophagoides *(*D*.)* farinae* crude extract was applied by the Okuda et al. method [14].

After these processes, AD symptoms such as lupus papules, hyperplasia of epithelial cells, subacute skin damage, and increased infiltration of lymphocytes, granular leukocytes, and degranulated mast cells were observed in NC/Nga mice [15]. Lipids of the stratum corneum are known to be responsible not only for cohesion among cells but also for functional regulation of the skin barrier [16]. We observed reduction of lipid distribution in the stratum corneum of the AE group based on Sudan black B staining.

After AD induction, mice in the AT group were orally given FFSO for 3 weeks daily. Animals were sacrificed 72 hours after the last* D. farinae* application, and histological specimens were collected.

Regarding H&E staining, atopic mice of the AE group exhibited significant damage to the intercellular space of the stratum corneum, hyperplasia, edema, infiltration of fibroblasts, and an increased capillary distribution. On the other hand, in mice treated with FFSO, remarkable reduction of histological skin lesions was observed. In the immunohistochemical results, p-ERK 1/2 as a cell activator showed increased expression in the AE group in the stratum granulosum. Expression of p-ERK 1/2 in the AT group decreased as compared with the AE group. Meanwhile, the neurotransmitter substance-P known to cause pain and pruritus showed increased expression, which might explain the psychological stress caused by aggravation of AD. Elevation of substance-P in tissues with AD is known to be connected with degranulation of mast cells, endothelium-dependent vasodilatation, transmission, and proliferation of inflammatory cells [17]. In the AT group, reduced numbers of positive cells for substance-P in the dermal papillae compared with the AE group were observed. Therefore, alleviation effects of FFSO in AD-induced skin lesions were observed in vivo.

Raw 264.7 cells were used to investigate the effects of FFSO in cells. Fc receptor expression, concentration of beta-hexosaminidase, nitric oxide assay, Western blotting, real-time PCR, image analysis, and statistical analysis were measured in cells. Immune response was determined by measuring cytokines secreted from differentiated helper T cells. Excessive differentiation of Th2 cells stimulated by increased secretion of IL-4, IL-5, and IL-6 leads to AD [18]. The Th2 cell response by IL-4 stimulation induces a specific reaction in the ligand-gated channel through the Janus family tyrosine kinase, which is a signal transducer and activator of the transcription (JAK-STAT) pathway [19]. Reduced distribution of IL-4-positive cells in the corium near the basal portion of the epithelium in the AT group was observed compared with the AE group. Therefore, we observed that FFSO could control cell differentiation by reducing IL-4- and STAT6-positive cells.

The transcription factor NF-*κ*B (nuclear factor kappa B), which induces expression of inflammatory and antiapoptotic genes, is known to cause tissue damage by accelerating expression of iNOS (inflammatory enzyme) [20, 21] and ICAM-1 (cell adhesion molecule) by inducing activation of LPS and proinflammatory cytokines such as IL-1*β* and TNF-*α* [22, 23]. In the AE group, distribution of NF-*κ*B p65 increased rapidly in the epithelial basal layer and papilla of the dermis, and the distribution of iNOS-positive cells in tissue increased gradually. The results show that FFSO treatment reduced the number of cells showing NF-*κ*B p65 and iNOS expression.

The protective effects of the lipid barrier were estimated by measuring LXR- and PKC-positive reactions, which are involved in lipid metabolism [16]. Levels of LXR in the AT group were higher as compared with the AE group, and elevation of PKC was significantly inhibited by FFSO treatment. Moreover, skin damage, such as elimination of the intercellular lipid lamellae in the stratum corneum, was remarkably reduced in the AT group as compared with the AE group.

## 5. Conclusion

In summary, FFSO can alleviate symptoms of AD such as epithelial damage, redness, swelling, and pruritus by recovering the protective function of the skin barrier and downregulation of inflammation.

## Figures and Tables

**Table 1:** Image analysis of immunohistochemistry in FFSO-treated mice after AD elicitation.

Objective	Group		
	CON	AE	AT
p-ERK 1/2	3,416 ± 63	44,068 ± 1013	17,601 ± 535^*∗*^
MMP9	639 ± 44	40,621 ± 813	8,329 ± 340^*∗*^
SP	1,367 ± 17	19,603 ± 655	3,934 ± 101^*∗*^
IL-4	1,767 ± 87	35,636 ± 910	10,304 ± 380^*∗*^
STAT6	251 ± 12	87,849 ± 1614	5,590 ± 178^*∗*^
NF-*κ*B p65	846 ± 22	472,211 ± 715	5,954 ± 110^*∗*^
iNOS	803 ± 26	11,200 ± 745	8,323 ± 696^*∗*^
5-HT	886 ± 32	65,375 ± 740	9,584 ± 581^*∗*^
TNF-*α*	1,183 ± 162	30,737 ± 1462	10,449 ± 1056^*∗*^
COX-2	1,251 ± 148	30,872 ± 1965	10,817 ± 1283^*∗*^
FC*ε* receptor	3,095 ± 261	39,508 ± 1136	16,637 ± 1227^*∗*^
LXR	132,645 ± 1920	2,704 ± 321	6,742 ± 269^*∗*^
PKC	994 ± 98	35,875 ± 2064	10,965 ± 1344^*∗*^
IL-6	1,412 ± 260	45,001 ± 2763	20,590 ± 1450^*∗*^
STAT3	973 ± 67	32,557 ± 1794	10,621 ± 1583^*∗*^

(Image analysis for 10,000,000 particles/range of intensity: 80–100.)

p-ERK: phosphorylated-extracellular-signal-related kinase 1/2; MMP-9: matrix metalloproteinases-9; SP: substance-P; IL-4: interleukin 4; STAT6: signal transducers and activators of transcription 6; NF-*κ*B p65: nuclear factor-*κ*B p65; iNOS: inducible nitric oxide synthase; 5-HT: serotonin; TNF-*α*: tumor necrosis factor-*α*; COX-2: cyclooxygenase 2; LXR: liver x receptor; PKC: protein kinase C; IL-6: interleukin 6; STAT3: signal transducers and activators of transcription 3; Ctrl: induced AD and given water; AE: induced AD and given water; AT: induced AD with FFSO treatment. ^*∗*^*P* < 0.05 compared with AE.
